# Artificial Intelligence Techniques and Health Literacy: A Systematic Review

**DOI:** 10.1016/j.mcpdig.2025.100269

**Published:** 2025-09-24

**Authors:** Abigail Naa Amankwaa Abeo, Sophie Armstrong, Michael Scriney, Hannah Goss

**Affiliations:** a School of Health and Human Performance, Dublin City University, Ireland; b School of Computing, Dublin City University, Ireland

## Abstract

**Objective:**

To systematically review the utilization of artificial intelligence (AI) in health literacy, highlighting limitations and future developments.

**Methods:**

A systematic review, following PRISMA guidelines, was conducted searching 6 databases for studies published from January 1, 2014, through April 10, 2024. Data extracted included population characteristics, health literacy definitions and measurement, study objectives, AI techniques, and metrics. Risk of bias was assessed using an adapted checklist.

**Results:**

From 1296 studies, 18 (1.4%) met inclusion criteria. These studies primarily evaluated text-based materials, including online articles, and electronic health records, with most materials in English, but also incorporated other languages. Artificial intelligence played various roles, including evaluating complexity, text simplification/readability enhancement, translation, and question-answering. Only 5 studies involved participant engagement. Seven studies provided a health literacy definition, consistently describing it as an individual’s ability to obtain, understand, and use health information for informed decisions, often linking it to external factors. However, only 1 study incorporated an individual level health literacy measurement tool, whereas organizational level health literacy measurement remained largely overlooked. The AI techniques used included traditional machine learning, deep learning, and transformer-based models. Evaluation metrics were categorized into human evaluation, readability, and machine learning metrics.

**Conclusion:**

The review highlights AI’s dynamic application in relation to health literacy; however, measurement of health literacy, at both an individual and organizational level, to evidence AI's effectiveness remains limited. In addition, future work should not only measure health literacy outcomes more rigorously but also pursue research on enhancing AI model performance, robust evaluation, and their practical implementation in real-world settings.

Health literacy is a modifiable determinant of health, influencing individuals’ ability to obtain, comprehend, assess, and apply health-related information.^1^ Although there are different definitions and interpretations of health literacy,^2^ the World Health Organization defines health literacy as “representing the personal knowledge and competencies that accumulate through daily activities, social interactions and across generations. Personal knowledge and competencies are mediated by the organizational structures and availability of resources that enable people to access, understand, appraise, and use information and services in ways that promote and maintain good health and well-being for themselves and those around them.”^3^ As a result of varied interpretations of the concept, there are different tools used to assess and monitor health literacy^4^ and various approaches to promoting health literacy globally.^5^ Yet, critically, there is consistent evidence for low levels of health literacy across the globe.^6^, ^7^, ^8^, ^9^, ^10^ Furthermore, there is a shared recognition that low health literacy can lead to negative health outcomes, reduced access to care, and poor disease control, and given these low rates globally, there is an urgent need to improve health literacy at both an individual level and organizational level internationally.^11^^,^^12^ Importantly, health literacy can be measured at these different levels. At the individual level, measurement tools capture a person’s ability to access, understand, and use health information. At the organizational level, measurement frameworks instead assess whether health information materials and systems are designed to be clear, accessible, and actionable for diverse audiences.^13^^,^^14^ Both approaches are relevant to evaluating AI’s role in health literacy; however, the distinction between these 2 levels is not always made explicit in the existing literature.

Recent advances in technology, and in artificial intelligence (AI) specifically, have opened new challenges and possibilities in relation to health literacy.^15^ The AI is a field of computer science that focuses on creating systems capable of performing tasks that typically require human intelligence.^16^ These tasks include learning from data, reasoning, problem solving, understanding natural language, recognizing patterns, and making decisions. Artificial intelligence can be categorized into various subfields, such as machine learning (ML; which enables systems to learn from data without explicit programming), natural language processing (which allows machines to understand and generate human language), and computer vision (which enables machines to interpret visual information).^16^^,^^17^ The study of AI and health literacy is evolving, with a small but growing number of studies exploring how AI can support access to, understanding of, and engagement with health information across diverse populations.^18^, ^19^, ^20^ For example, AI has been used in health literacy through chatbots built on large language models to provide educational support and enhance understanding of medical topics like vaccination^21^ and to automatically identify and curate understandable and medically relevant health information, such as YouTube videos on COVID-19.^22^ It has also been used to assess and alter the readability of online health resources.^23^ It is important to note that although AI offers potential for improving health literacy, according to Nutbeam,^15^ this potential has not yet been extensively evaluated or actualized. In addition, at this stage, concerns exist regarding the accuracy, completeness, bias, and potential for misinformation and disinformation.^7^^,^^18^^,^^24^ Hence, it is often recommended that AI interventions be complemented with human supervision and oversight to ensure the delivery of accurate and reliable information^25^^,^^26^; yet, it is somewhat unclear whether and how this is done in practice across the field.

Given the lack of conceptual clarity across both AI and health literacy, the fragmented research across disciplines, and the limited evidence supporting this emerging application at the intersection of AI and health literacy, there is a need for a systematic review of existing studies in this area. Findings of such a review would help understand the state of the science and inform a more systematic approach to progressing research in this field. Specifically, this review aims to answer the following research questions (RQ):

1. In studies that have incorporated the use of AI, how has health literacy been defined and what health literacy measurement tools have been used, with consideration of health literacy as both an individual and organizational concept?
2. What AI techniques have been used in the field of health literacy and how have they been used?
3. Within these studies, what metrics have been used to evaluate the AI techniques?

## Methods

A systematic review of which ML approaches have been applied in studies related to health literacy in the last decade was conducted in accordance with the Preferred Reporting Items for Systematic Reviews and Meta-Analyses (PRISMA) 2020 guidelines^27^ to ensure the transparent and high-quality reporting of reviews (Supplemental Table 1, available online at https://www.mcpdigitalhealth.org/). The protocol for this review was registered with PROSPERO (CRD42024544276).

### Eligibility Criteria

We included studies that related to the exploration of health literacy and/or the enhancement or improvement in health literacy (this could be direct or indirect). We considered studies identified by the search if they reported on ML-related techniques, for example (but not limited to), neural networks, natural language processing, and logistic regression in relation to health literacy. Studies were excluded if they were published in a non-English language; if they were editorials, systematic reviews, commentaries, and duplicate publications; or if full-text articles were not available.

### Information Sources and Search Strategy

Six databases were searched, namely, Scopus, CINAHL, ERIC International, PubMed, Web of Science, and MEDLINE for articles published between January 1, 2014, and April 10, 2024. This timeframe was selected in line with advances in AI and informed through initial scoping searches. The broad selection of databases was used to ensure extensive and comprehensive coverage of applicable literature.

The main keywords identified for this study were “health literacy” and “machine learning.” Other phrases, keywords, and acronyms similar or related to these keywords were also identified, such as “health education,” “health promotion,” and “artificial intelligence.” Search terms were combined using Boolean operators (AND/OR) to construct precise search queries and refine and narrow the search results (Supplemental Appendix 1, available online at https://www.mcpdigitalhealth.org/).

### Selection Process

First, all titles and abstracts were screened independently using Covidence^28^ by 2 reviewers (A.N.A.A and S.A.) to assess their relevance based on the predefined inclusion and exclusion criteria. In cases where disagreements arose, the conflict was resolved through discussion between the initial reviewers and consultation with other authors (H.G. and M.S.). This approach ensured that all decisions regarding this stage were thoroughly examined and agreed upon by a majority of the review team. During the second stage of full-text screening, a single reviewer (A.N.A.A) assessed the articles for eligibility. In instances of uncertainty or ambiguity, the reviewer consulted other authors (H.G. and M.S.).

### Data Extraction

Data extraction (completed by A.N.A.A.) captured: article information (author(s), year of publication, title, journal, and country of first and last authors’ affiliated institution), characteristics of the study (objective/aim and any population characteristics), data analysis/methods (AI approach used, data types, and performance metrics used), results (health literacy measurement tools used, performance metrics, and limitations), and risk of bias assessment. This information is presented through narrative synthesis.

### Risk of Bias Assessment

Risk of bias was assessed following a previously developed checklist from Wen et al^29^ and Malhotra^30^ to assess the rigorousness, credibility, and relevance of the relevant studies. This quality checklist was adapted for the current review to include 3 additional questions (Q5, Q13, and Q14) to address critical aspects of health literacy (Table 1). The checklist questions are rated as follows: 1 (yes), 0.5 (partly), 0 (no), and 0 (not reported), with a total score calculated by summing the values provided to each question. A study may have a maximum score of 14 and a minimum score of 0. The scores were categorized as follows: very high (11 ≤ score ≤ 14), high (9 ≤ score ≤ 10.5), medium (6 ≤ score ≤ 8.5), and low (0 ≤ score ≤ 5.5). The assessment was done by a single reviewer (A.N.A.A), with uncertainties resolved through discussions with other authors (H.G. and M.S.).

**Table 1:** Risk of Bias Questions

Q#	Question
Q1	Are the aims of the research clearly stated?
Q2	Are the independent variables clearly defined?
Q3	Is the data set size appropriate?
Q4	Is the data collection procedure clearly defined?
Q5	Are participants involved in the study for evaluation purposes?
Q6	Are the ML techniques clearly defined?
Q7	Are the ML techniques justified?
Q8	Are the performance measures used to assess the ML models clearly defined?
Q9	Are the results and findings clearly stated?
Q10	Are the limitations of the study specified?
Q11	Is the research methodology repeatable?
Q12	Is there any comparative analysis conducted (ML vs ML)?
Q13	Is health literacy defined?
Q14	Are the measures used to measure health literacy defined?

ML, machine learning.

## Results

### Search Results

**Figure 1:**
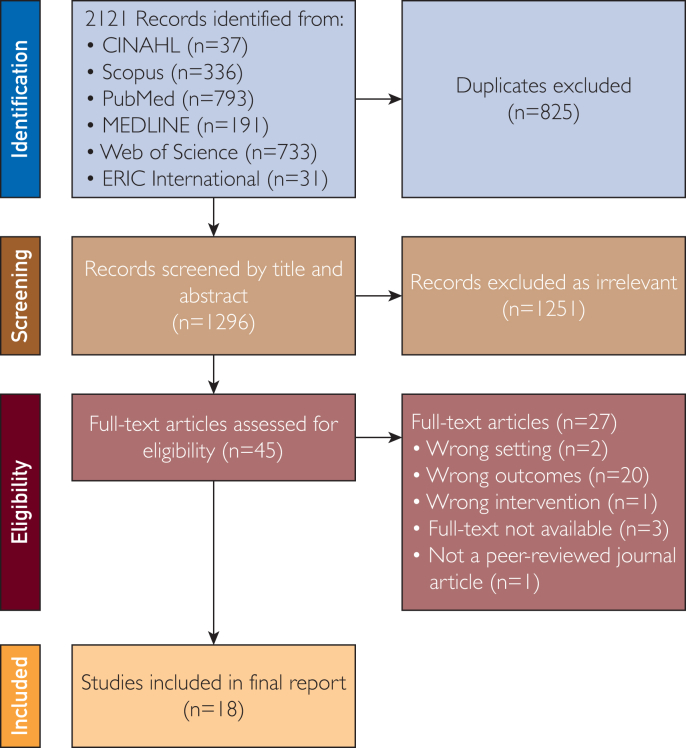
Preferred Reporting Items for Systematic Reviews and Meta-Analyses (PRISMA) flow diagram.

Our systematic search yielded a total of 2121 potentially relevant records across all databases. Following duplicate removal using Covidence, 1296 unique records were retained. After the title and abstract screening, 45 studies met the inclusion criteria for full-text review. Of these, 18 studies were deemed eligible and incorporated into the systematic review. A detailed flow diagram outlining the identification and screening process is presented in Figure 1.

### Study Characteristics

**Figure 2:**
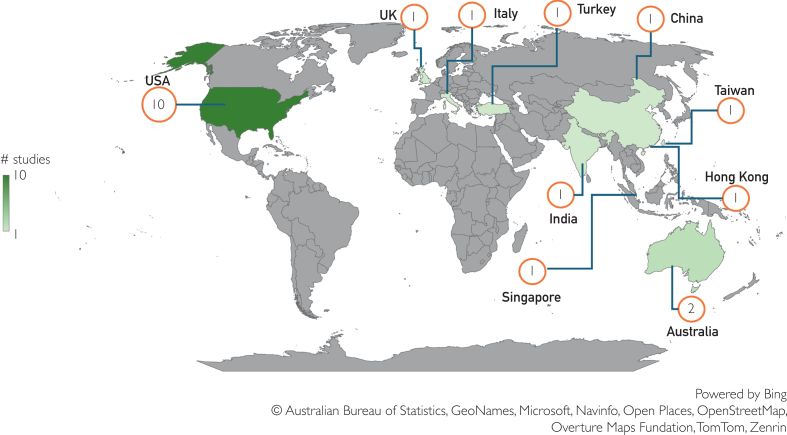
Origin of studies. Note that the first and last authors had different affiliations in 2 articles. The numbers on the map thus sum to 20 rather than 18 (total number of included studies).

We undertook a systematic review, applying a structured and transparent process to identify, screen, and analyze studies on AI and health literacy. Although the 18 included studies vary in design and focus, their diversity is valuable: it illustrates both the range of AI applications being tested and the current gaps in this emerging field. Figure 2 shows the origin of the included studies based on the institutional affiliations of the first and last authors. The included studies evaluated a wide range of materials and data sources, primarily in text-based formats. These included patient-facing educational resources such as online articles,^31^, ^32^, ^33^ webpages,^7^^,^^34^, ^35^, ^36^, ^37^ frequently asked questions,^18^^,^^38^^,^^39^ patient information leaflets,^11^ informed consent forms,^25^ and electronic health records (EHRs).^34^ Clinical documents^40^ and secure patient-physician messages^12^ were also analyzed for tasks such as information extraction, and simplification. Additionally, structured medical knowledge bases^11^^,^^34^ and annotated datasets^11^^,^^12^^,^^35^^,^^36^^,^^38^^,^^40^ were used for model development and evaluation. In terms of language, most materials were in English, but several studies incorporated other languages to support multilingual health communication. These included Italian,^11^ Spanish,^31^^,^^38^ Chinese,^38^^,^^39^ Hindi,^41^ Malay,^38^ Tamil,^38^ Filipino,^38^ Thai,^38^ Japanese,^38^ French,^38^ and Portuguese.^38^ This reflects a growing emphasis on inclusivity in AI-driven health literacy materials.

We categorized the included studies based on the specific roles AI played in relation to health literacy. Seven studies^7^^,^^12^^,^^25^^,^^34^, ^35^, ^36^^,^^40^ examined the use of AI to evaluate the complexity of health information. Text simplification and readability enhancement, aimed at producing accessible materials, was the focus of 9 studies.^7^^,^^25^^,^^31^, ^32^, ^33^, ^34^^,^^40^, ^41^, ^42^ Artificial intelligence–driven translation of health content to other languages appeared in 2 studies.^38^^,^^41^ A total of 6 studies^11^^,^^18^^,^^26^^,^^34^^,^^37^^,^^39^ involved AI systems designed to answer health-related questions and provide interactive interventions. These findings help map the functional landscape of AI applications and highlight areas for further development.

Of the 18 included studies, only 5 (27.8%) involved participant engagement,^11^^,^^34^^,^^37^, ^38^, ^39^ with sample sizes ranging from 28 to 109. Detailed demographic information for these studies is provided in Supplemental Table 2 (available online at https://www.mcpdigitalhealth.org/). Among the included studies, 3 of them focused on surgical disciplines, including orthopedic surgery,^33^ and pediatric urology.^26^ Other areas examined included hypertension,^18^ diabetes,^12^ and radiology.^41^ Disciplines such as orthopedic surgery and diabetes mostly target medical practitioners as their intended users, whereas the others are predominantly oriented toward patients, except for radiology, which caters to both groups. Collectively, these studies highlight the diverse applications of health literacy research across medical disciplines. Table 2 provides an overview of the key characteristics and findings of included studies.

**Table 2:** Overview of Included Studies

S. No.	Reference, year	Country	Objective	AI technique	Performance metrics	Key findings
1.	Ali et al,^25^ 2024	USA	To evaluate the GPT-4 general LLM’s ability to simplify surgical consent forms, establish a streamlined framework, and generate comprehensible, procedure-specific consents	GPT-4	Median (number of characters and words, characters per word, words per sentence), reading time, average rarity of words, FKRL, and FRES	• Improved readability metrics significantly (reading level, 13.9-8.9; reading ease, 35-64; all *P*<.01). • De novo consent forms written at sixth-grade level, scored 20/20 on a validated rubric, and passed expert surgeon review.
2.	Almagazzachi et al,^18^ 2024	USA	To assess ChatGPT’s accuracy and reproducibility in responding to frequently posed inquiries on hypertension	ChatGPT	Reproducibility and accuracy	• Accuracy: 92.5% appropriate (7.5% inappropriate); validated against guidelines+physician review. Reproducibility: 93% consistent answers.
3.	Baldwin,^7^ 2024	UK	To assess the potential of an AI language model in enhancing the readability of online first aid burn material	ChatGPT-3	FRES, FKGL, GFI, CLI and SMOG	• Readability: Target-level webpages improved from 4% to 18%. All metrics found significant improvement (FRES, 73.5 → 82.1; FKGL, 5.9 → 4.9; GFI, 8.2 → 7.4; CLI, 8.3 → 6.9; SMOG, 6.1 → 5.3; all *P*<.001).
4.	Caglar et al,^26^ 2024	Turkey	To assess the accuracy and reliability of ChatGPT’s answers to commonly asked questions concerning pediatric urology	ChatGPT	Accuracy and reproducibility	• Accuracy: 92.0% (grade 1 completely correct), 5.1% (correct but insufficient), 2.9% (partly correct+misleading), 0% completely wrong. Reproducibility: 93.8%-100% consistency across questions. • No completely incorrect answers, but 3%-5% responses insufficient or potentially misleading, underscoring need for expert oversight.
5.	Chang et al,^39^ 2023	Taiwan	To develop an interactive health care question-answering robot that provides real-time responses from a preexisting knowledge base and recommends supplementary pertinent questions and answers	SBERT, RoBERTa, BERT, T5, and MiniLM	Precision at 1 (P@1), mean reciprocal rank at 5, normalized discounted cumulative gain at 5	• Accuracy: Outperformed BM25 and other baselines, with ≥12% gain in P@1. • User study: High ratings (4.33±0.59) on understandability, relatedness, effectiveness, and engagement.
6.	Crossley et al,^12^ 2020	USA	To develop an automated readability technique to better grasp which components of physicians’ digital messages contribute to their clarity or obscurity	LDA	Accuracy, sensitivity, specificity, and confusion matrix	• Accuracy: MoTeR-P, 74.9% (sensitivity=0.674; specificity=0.788); vs FKGL, 65.0% (sensitivity=0.302; specificity=0.838). • MoTeR-P better aligned with expert ratings, capturing lexical sophistication, cohesion, and sentiment features.
7.	Doppalapudi et al,^40^ 2022	USA	To create an NLP pipeline that can extract pertinent information from extensive unstructured clinical notes and simplify lexicons by substituting medical jargons and technical terminologies	LSTM, SEQ, and BioBERT	F1 score, accuracy, FKRL, GFI, CLI, BLEU score, and SARI	• Accuracy: Diagnostic information extraction F1 score=49%-51%; microaccuracy=68.7%-71% (close to CNN benchmarks). • Readability: Simplification improved grade levels (GFI, 14.2→8.8; FKGL, 7.1→5.1; CLI, 12.0→6.5). • Meaning preservation: BLEU=79.9; SARI=27.7.
8.	Hendawi et al,^34^ 2022	USA	To formulate, implement, and assess a mobile health application, MediReader, to enhance individuals’ comprehension of difficult medical content and enhance their health literacy	BiLSTM-CNN-CRF neural network, and KNN	Likert scale responses	• Experimental users scored 76% vs 36% (control). • Usability: 85% reported improved understanding, 78% ease of use, and 85% would recommend.
9.	Ji et al,^35^ 2021	Australia/China	To investigate multidimensional semantic attributes to construct machine learning algorithms that can predict the estimated degree of cognitive access of English health information	LogitBoost, SVM, decision tree classifier, and logistic regression	AUC, sensitivity, specificity, and accuracy	• Best model LogitBoost AUC, 0.858; accuracy, 0.802; sensitivity, 0.787; specificity, 0.813. • SVM second best (AUC, 0.848; accuracy, 0.786). • Semantic features (logical structure, lexical familiarity, and abstractness) more predictive of accessibility than word/sentence length.
10.	Ji et al,^36^ 2021	Australia/Hong Kong	To develop machine learning algorithms that assess the comprehensibility of English health materials for non-English speaking tertiary students by integrating internationally recognized clinical guidelines	XGBoost, random forest, multilayer perceptron, decision tree, and logistic regression	AUC, sensitivity, specificity, and accuracy	• Best models were decision tree and XGBoost: accuracy, 0.945 both; AUC, 0.981/0.979; sensitivity, 0.950/0.947; specificity, 0.941/0.944, respectively. • Text clarity, logical sequence, and educational relevance matter more for understandability than domain knowledge or numeracy for the international students with moderate English proficiency but high health literacy.
11.	Kirchner et al,^33^ 2023	USA	To investigate whether an AI dialog platform can revise orthopedic patient education materials to decrease reading levels from high school to sixth-grade, while maintaining accuracy and adequate content detail for instructive purposes	ChatGPT	FRES and FKGL	• FKGL reduced from grades 9.5-12.6 to 5-6 (≈47%-56% reduction). FRES improved to 75-82 (easier readability). • Stable results on repeated trials. No factual errors detected; revised materials retained sufficient detail for patient education.
12.	Mane et al,^37^ 2023	USA	To develop and refine a question and answering chatbot called Rosie to support the health of pregnant and new mothers of color	Contriever	Accuracy	• 98.5% accuracy in correctly identifying user intents (eg, greetings, health questions, and emergencies). Contriever retrieval preferred 66% over dense passage retrieval baselines. • Usability: Community survey (n=109): 96% likely to use; top queries on baby development and nutrition.
13.	Minutolo et al,^11^ 2022	Italy	To develop a conversational agent that makes the health information encoded in Italian Patient Information Leaflets easily accessible	BERT and TED	F1 score, accuracy, dialog success rate, and questionnaires	• Accuracy, 98.3%; F1 score, 96.8%. • Usability: Positive ratings on questionnaires (good–excellent user experience).
14.	Rouhi et al,^32^ 2024	USA	To determine if 2 accessible generative AI dialog platforms can revise online aortic stenosis (AS) patient education materials (PEMs) to align with the suggested reading proficiency levels	ChatGPT-3.5 and BARD	FRES, FKGL, GFI, and SMOG	• Both ChatGPT-3.5 and Bard significantly improved readability of aortic stenosis PEMs. • ChatGPT-3.5 outperformed Bard across all readability metrics, with faster conversion times and better alignment to the recommended approximately sixth-grade level, although neither platform consistently achieved below or equal to the sixth-grade benchmark.
15.	Sarangi et al,^41^ 2023	India	To evaluate the capacity of ChatGPT to reconcile technical radiological terminology with simple dialect, to improve comprehension as well as involvement for both medical professionals and patients	ChatGPT-3.5	Likert scale and consistency	• Accuracy: Expert ratings 85%-94% across English reports. Strong interrater agreement (ICC=0.873). • ChatGPT-3.5 simplified English radiological reports effectively, reducing jargon and maintaining diagnostic accuracy. • Performance in Hindi translation was poor, with incomplete and grammatically incorrect outputs.
16.	Sudharshan et al,^31^ 2024	USA	To assess whether physicians can endorse ChatGPT-3.5 for patients to simplify ophthalmological texts in both English and Spanish	ChatGPT-3.5	FRES, GFI, FKGL, Fernández Huerta (FH), Gutiérrez, Szigriszt-Pazos (SP), INFLESZ, Legibilidad-μ (Lμ), and Crawford Nivel-de-Grado (CNG)	• English texts: ChatGPT-3.5 did not significantly improve readability. • Spanish texts: Significant improvements only seen in CNG and Lμ indices; other Spanish metrics found no significant change.
17.	Vallurupalli et al,^42^ 2024	USA	To examine the effectiveness of ChatGPT 3.5 in simplifying and evaluating the readability of educational materials on craniofacial surgery for patients	ChatGPT-3.5	FRES, GFI, FKGL, CLI, SMOG, automated readability index, Linsear write formula, ChatGPT 3.5’s internal assessment, and accuracy	• ChatGPT 3.5 performed comparably with traditional readability scores for baseline assessment and significantly simplified craniofacial patient materials to within recommended grade levels.
18.	Yang et al,^38^ 2023	Singapore	To develop and evaluate a multilingual chatbot for accurately answering open-ended COVID-19 questions	BERT	Accuracy, AUC, precision, recall, and F1 score	• DR-COVID achieved high accuracy (83.8%) with strong AUC (91.7%) and F1 (82.9%) scores on the English data set. • However, accuracy decreased when tested on novel external questions, highlighting the importance of data set coverage, and ongoing retraining. • Multilingual support was promising (best in Portuguese and weaker in French).

AI, artificial intelligence; AUC, area under the receiver operating characteristic curve; BARD, Google's BARD model; BLEU, bilingual evaluation understudy; CLI, Coleman-Liau Index; CNN, convolutional neural network; CRF, conditional random field; FKGL, Flesch-Kincaid Grade Level; FRES, Flesch Reading Ease Score; GFI, Gunning Fog Index; KNN, K-nearest neighbor; LDA, linear discriminant analysis; LSTM, long short-term memory; LLM, large language model; MoTeR-P, Model of Text Readability in Physicians; NLP, natural language processing; SARI, system output against references and against the input sentence; SEQ, Sequence Labeling Model; SMOG, Simple Measure of Gobbledygook; TED, transformer embedding dialogue.

### RQ1: In Studies That Have Incorporated the Use of AI, How Has Health Literacy Been Defined and What Health Literacy Measurement Tools Have Been Used?

From the studies included, 7 provided definitions of health literacy.^7^^,^^11^^,^^12^^,^^26^^,^^34^^,^^40^^,^^42^ Across the definitions, several common characteristics emerged. Health literacy is consistently described as an individual’s ability to obtain, understand, and use health information to make informed decisions. Many definitions highlight the role of comprehension, communication, and decision making in navigating health care. Additionally, health literacy is often linked to external factors such as education level, socioeconomic status, access to resources, and cultural influences. Table 3 provides an overview of the 7 definitions of health literacy provided in the included studies. Among the studies included in this review, only 1 used a health literacy measurement tool.^34^ This study used ComprehENotes,^43^ a tool designed to assess individual health literacy levels by leveraging questions derived from real patient EHRs.^34^

**Table 3:** Health Literacy Definitions

Reference, year	Definitions
Baldwin,^7^ 2024	“The personal characteristics and social resources necessary for individuals and services to make informed decisions about health.”
Caglar et al,^26^ 2024	“Health literacy encompasses a patient’s ability to comprehend and interpret medical information and subsequently take appropriate actions.”
Crossley et al,^12^ 2020	“Health literacy can include a number of demographic and individual difference factors including education level, culture, access to resources, socioeconomic status, and age, among others. HL also includes a patient’s ability to obtain, process, comprehend, and communicate basic health information and is highly correlated with literacy skills.”
Doppalapudi et al,^40^ 2022	“Personal health literacy is about an individual’s ability to find, understand, and use information for health-related decisions and actions. Organizational health literacy concerns the degree to which organizations enable individuals to enforce personal health literacy.”
Hendawi et al,^34^ 2022	“The degree to which individuals have the capacity to obtain, process, and understand basic health information and services needed to make appropriate health decisions.”
Minutolo et al,^11^ 2022	“Health literacy is an emerging concept built on the idea that both health and literacy are critical resources for everyday living.” “Health literacy is also connected with the concept of self-efficacy and “self-empowerment”, introducing the idea that, by improving health literacy, individuals will be able to read better the information leaflet given by their doctor, take their medication as prescribed, and, thus, they will also be empowered to take a more participative and self-confident role in their healthcare.”
Vallurupalli et al,^42^ 2024	“Personal health literacy is the degree to which individuals have the ability to find, understand, and use information and services to inform health-related decisions and actions for themselves and others.”

### RQ2: What AI Techniques Have Been Used in the Field of Health Literacy and How Have They Been Used?

#### Traditional ML Models in Health Literacy

Four studies^12^^,^^34^, ^35^, ^36^ applied ML techniques to evaluate the readability and accessibility of health texts and to improve the linking of medical terms in health information systems. One study developed the Model of Text Readability in Physicians, which predicts expert ratings of text complexity in medical documents. This was achieved using linear discriminant analysis.^12^ Another study applied the K-nearest neighbor algorithm within the MediReader application to generate candidate entities for linking a given mention, with the aim of supporting information retrieval.^34^ Two studies used a range of classification models—including decision trees, random forests, and logistic regression—to assess the cognitive accessibility and linguistic clarity of English language health materials aimed at international tertiary students.^35^^,^^36^ (See Glossary in Supplemental Table 3, available online at https://www.mcpdigitalhealth.org/.)

#### Deep Learning Models in Health Literacy

Four studies^34^^,^^36^^,^^37^^,^^40^ used deep learning models in health literacy, applying them to tasks such as text classification, complex word identification, medical entity recognition, and readability classification. Long short-term memory (LSTM)^44^ network was used for multilabel classification, mapping information extracted from clinical notes to patients’ diagnosis codes.^40^ The Sequence Labeling Model^45^ was applied to identify complex words within clinical text, with the aim of facilitating improved readability and comprehension.^40^ A hybrid deep learning model, BiLSTM-CNN-CRF,^46^^,^^47^ which integrates bidirectional long short-term memory (BiLSTM), convolutional neural networks (CNNs), and conditional random fields (CRFs), was used for medical entity identification in the MediReader mobile health application.^34^ Additionally, a Multilayer Perceptron was used to predict how understandable health texts were, for international college students, with the goal of aiding in the development of materials that are more accessible to diverse populations.^36^ Moreover, the RASA framework^48^ was used to build Rosie, a chatbot designed to support pregnant women and new mothers of color by classifying user queries and retrieving relevant health information.

#### Transformer-Based Models in Health Literacy

Six studies^11^^,^^25^^,^^37^, ^38^, ^39^, ^40^ have applied transformer-based models in health literacy, with 4 studies^11^^,^^38^, ^39^, ^40^ specifically using bidirectional encoder representations from transformers (BERT) and its variants. Additionally, 8 studies^7^^,^^18^^,^^26^^,^^32^^,^^33^^,^^37^^,^^41^^,^^42^ have investigated their application in conversational AI models and chatbots, predominantly concentrating on ChatGPT and its variants. These models were applied for tasks such as text simplification, question-answering, medical entity extraction, and conversational AI, aiming to improve accessibility and information retrieval in health-related contexts.

GPT-4 (Generative Pretrained Transformer 4)^49^ was used to simplify surgical consent forms, with the aim of ensuring that patients can better understand critical medical documents.^25^ Text-to-Text Transfer Transformer^50^ was applied for question generation, with the aim of creating diverse queries to enhance health information retrieval.^39^ Contriever^51^ was integrated into Rosie, a question-answering chatbot designed for pregnant women and new mothers of color, with the goal of assisting in retrieving relevant responses from the system’s knowledge base.^37^ The TED (Transformer Embedding Dialog)^52^ model was used in dialog action selection, with the aim of refining chatbot responses to user queries.^11^ The MiniLM^53^ was used as a question filtering model, with the aim of removing inappropriate or irrelevant queries to maintain the accuracy and reliability of chatbot interactions.^39^

The BERT model^54^ was used in several ways. In 1 study, it ranked frequently asked questions based on relevance.^39^ In another, it acted as the natural language interpreter in a conversational agent for Italian, helping the system understand user intent and fill in missing details.^11^ A locally adapted version of BERT was used in the multilingual DR-COVID chatbot for answering COVID-19 questions.^38^ Other variants of BERT had more specialized roles: BioBERT^55^ was used to suggest simpler alternatives for biomedical terms^41^; RoBERTa (Robustly Optimized BERT Pretraining Approach)^56^ was used to identify medical-related terms^39^; and SBERT^57^ supported semantic search and question encoding to improve information retrieval.^39^

ChatGPT was used to provide accessible knowledge on hypertension^18^; generate responses to pediatric urology questions^26^; and rewrite orthopedic education materials at a sixth-grade level.^33^ ChatGPT-3 was applied to simplify text to a readability level suitable for an 11-year-old child, while maintaining an adult-appropriate tone.^7^ ChatGPT-3.5 was widely used to rewrite patient materials on aortic stenosis,^32^ simplify radiology reports,^41^ and craniofacial texts in English and Spanish.^42^

### RQ3: Within These Studies, What Metrics Have Been Used to Evaluate the AI Techniques?

Across the included studies, 3 outcomes were consistently evaluated: accuracy and content quality, readability, and AI model performance. First, human assessment was central to evaluating accuracy and content quality (n=7). In 4 of these studies,^18^^,^^26^^,^^33^^,^^42^ accuracy was verified by authors, specialists, or comparing outputs to clinical guidelines. Reproducibility was addressed in 2 studies,^18^^,^^26^ and Likert scale ratings to capture subjective perceptions were applied in 2 studies.^34^^,^^41^ One study^11^ used both questionnaires and dialog success rate to evaluate user experience, whereas another^41^ assessed consistency of outputs.

Machine learning metrics (simple tools or numbers used to measure how well an AI or ML model is doing) were used in 8 studies to evaluate AI models. Accuracy was the most used metric, found in 7 of these studies^11^^,^^12^^,^^35^, ^36^, ^37^, ^38^^,^^40^ followed by sensitivity (4 studies),^12^^,^^35^^,^^36^^,^^38^ F1 score (3 studies),^11^^,^^38^^,^^40^ specificity (3 studies),^12^^,^^35^^,^^36^ and area under the curve (3 studies).^35^^,^^36^^,^^38^ One study^12^ used a confusion matrix to analyze classification performance. Ranking and retrieval metrics such as Precision@1, Mean Reciprocal Rank@5, and Normalized Discounted Cumulative Gain@5 were used in 1 study.^39^ BLEU and SARI scores, relevant for evaluating text generation and simplification, were used in a study.^40^

Finally, 7 studies assessed the linguistic complexity and accessibility of health information using various readability metrics. The Flesch-Kincaid Grade Level and Flesch Reading Ease Score were the most frequently used, each appearing in 6 studies.^7^^,^^25^^,^^31^, ^32^, ^33^^,^^42^ The Gunning Fog Index was used in 5 studies,^7^^,^^31^^,^^32^^,^^40^^,^^42^ while the Simple Measure of Gobbledygook and Coleman-Liau Index (CLI) were each applied in these 3 studies.^7^^,^^32^^,^^42^ One study^25^ used additional readability metrics including character and word counts, reading time, sentence length, and passive voice usage. Another study^42^ used the Automated Readability Index, Linsear Write Formula, and ChatGPT-3.5’s internal assessment. Spanish readability indices—such as Fernández Huerta, Szigriszt-Pazos, INFLESZ, Legibilidad-μ, and Crawford Nivel-de-Grado—were used in 1 study.^31^

### Risk of Bias Assessment

Among the included studies, 2 (11%) were rated as very high quality^12^^,^^34^; 11 (61%) as high^7^^,^^11^^,^^26^^,^^32^^,^^35^, ^36^, ^37^, ^38^, ^39^, ^40^^,^^42^; and 5 (28%) as medium^18^^,^^25^^,^^31^^,^^33^^,^^41^; none received a low rating (Supplemental Table 4, available online at https://www.mcpdigitalhealth.org/) based on the adapted study quality assessment. Some criteria the studies performed inadequately on were Q5 (Are participants involved in the study for evaluation purposes?), Q12 (Is there any comparative analysis conducted (ML vs ML)?), Q13 (Is health literacy defined?), and Q14 (Are the measures used to measure health literacy defined?).

## Discussion

Our review of the studies reveals a dynamic field where AI is being increasingly applied to health literacy across a variety of areas. This landscape is characterized by the deployment of diverse AI techniques, each with its own strengths and limitations. The studies included within this review highlight a range of AI methodologies including traditional ML models, deep learning models, and transformer-based models. Transformer-based models, such as BERT and GPT-4, are prominent for tasks such as text simplification, question-answering, medical entity extraction, real-time, tailored responses, and interactive access to health information, potentially empowering individuals in their health management.^58^^,^^59^ Deep learning models, including LSTM and BiLSTM-CNN-CRF, are also used for sophisticated tasks such as text classification and readability prediction. Although these models excel at processing complex, unstructured data and identifying long-term dependencies, they do demand significant computational resources.^60^^,^^61^ In contrast, traditional ML methods, such as linear discriminant analysis and K-nearest neighbors, are easier to understand and require less computing power. This makes them useful for simpler tasks, like checking how easy a text is to read or linking medical terms in structured data sets.^62^^,^^63^ Within this review, all these different techniques are being applied across various fields, demonstrating the widespread interest in leveraging AI to improve the accessibility and understanding of health information. This aligns with findings from a recent systematic review by Nasra et al,^64^ which synthesized evidence across multiple domains and highlighted the increasing application of AI, particularly, large language models such as ChatGPT, to improve the clarity of patient educational materials. The expanding role of AI in this context underscores its potential to transform health communication and support health literacy, particularly over the past decade.

According to Doppalapudi et al^40^ and Sarangi et al,^41^ one of the primary challenges in health care is the complexity of medical documents, which often contain technical jargons and specialized terminology, which can be difficult for nonexperts to comprehend. Various studies within this review^25^^,^^40^^,^^41^ highlight the use of AI to simplify complex medical information, including radiological reports, surgical consent forms, and patient education materials. With the goal of enhancing understanding for both health care professionals and patients, these applied studies address the challenge of translating technical jargons in these documents to language more suitable to the public. Further research included in this review^7^^,^^32^^,^^33^ indicate that AI can rephrase materials and health information to lower reading skills levels while retaining accuracy. This can be achieved by using simple prompts such as “translate to 5th-grade reading level” or “rewrite this paragraph for an 8th grader.”^32^^,^^33^^,^^42^ Throughout the included studies within this review, the use of ML techniques to simplify and rewrite medical information has been found, but researchers need to be conscious of accuracy and interpretability, as well as computational cost.

Multiple papers have highlighted the importance of transparently presenting a definition^2^ because this has implications for assessment, for comparison of results, and for the development of best practice—in this context with regards to using AI in relation to health literacy. Health literacy was frequently defined by included studies as an individual’s capacity to discover, comprehend, and use health information to make informed decisions.^7^^,^^11^^,^^12^^,^^26^^,^^34^^,^^40^^,^^42^ Doppalapudi et al,^40^ however, also addressed organizational health literacy, which emphasizes how institutions and health care systems play a role in ensuring that health information is accessible, understandable, and actionable for diverse populations. This acknowledgment of health literacy as both a personal and organizational construct is increasingly common in the HL field, and it is encouraging to see this reflected within this cross-disciplinary area.

Despite the growing focus on the potential of improving health literacy through AI, the measurement of health literacy to evidence the effectiveness of these approaches appears limited within studies included in this review for outcomes such as comprehension, accessibility, decision making, confidence, and communication. Only 1 study^34^ included an assessment of health literacy at the individual level. The authors of this tool tackle an issue: the potential for patients with limited health literacy to struggle with understanding their EHR notes.^43^ This is perhaps reflective of early stage of the field, and the health literacy research more broadly, which has debated the definition, and subsequent evaluation of the concept across different populations.^65^^,^^66^ In contrast, organizational health literacy measurement—which examines the extent to which health systems, services, and tools are designed to make health information clear, accessible, and actionable^14^—has received little attention in the included studies. Within the health literacy field more broadly, there has been a growing recognition of the mediating influence of organizational structures and available resources on an individual’s health literacy.^67^ In relation to the application of AI for health literacy, consideration of organizational health literacy could, for example, improve the design and content of online materials. However, as such a novel and rapidly emerging area, more research is needed to establish organizational health literacy principles to best support the design and use of AI models. Subsequently, without valid, reliable, and feasible health literacy measurement tools, at both an individual level and perhaps more importantly an organizational level, it becomes difficult to assess how effective health literacy interventions are: in this case, AI interventions and how to best support the development of health literacy. In relation to AI supported interventions specifically, such information could improve the focus, feasibility, and effectiveness of this technology and, ultimately, health literacy outcomes.

Within this review, a variety of metrics were used to evaluate the use of AI techniques in health literacy. This is reflective of the wider health literacy and AI fields. Human assessment played a crucial role in many of the included studies,^18^^,^^26^^,^^33^^,^^34^^,^^41^^,^^42^ with accuracy checks by “experts” (such as authors and physicians) and the use of Likert scale responses being common methods to evaluate the clarity and effectiveness of AI outputs. A wide array of ML metrics such as accuracy, sensitivity, and F1 score were used to quantify the performance of AI models, with the specific metric often depending on the AI model or technique used.^11^^,^^12^^,^^35^^,^^36^^,^^38^, ^39^, ^40^ Numerous readability metrics, including the Flesch-Kincaid Reading/Grade Level and the Gunning Fog Index, were used to assess the complexity and accessibility of health-related texts simplified or generated by AI.^7^^,^^25^^,^^31^, ^32^, ^33^^,^^42^ Nutbeam^15^ argues that at this juncture in the development of AI platforms, substantial human expertise and discernment are essential. As such, it is encouraging to see many of these studies included some form of human expert interaction/assessment. The heterogeneity in different approaches, however, highlights the need to identify best practice methods to ensure the use of AI techniques in health literacy is accurate, reliable, trustworthy, and responsive.

Developing the usability and effectiveness of digital health tools, such as mobile applications and conversational agents, requires participant involvement, beyond traditionally viewed experts. For example, the MediReader mobile application study^34^ involved observing participants’ interactions and collecting Likert scale responses, while the patient information leaflet chatbot^11^ incorporated a blend of questionnaires to evaluate performance and usability. Community-engaged testing, such as with the Rosie chatbot,^37^ was essential for gathering feedback on design, content, and cultural relevance. Participants evaluated the interactive health care robot’s relatedness, effectiveness, and engagingness.^39^ These factors are dependent on the intended audience, and as such, those involved in the development and evaluation of digital health tools should be reflective of the intended audience. Ultimately, a combination of both experts and intervention users are needed to improve the accuracy, reliability and feasibility of the application of AI to improve health literacy.

### Limitations of Included Studies

Numerous studies^7^^,^^11^^,^^25^^,^^31^, ^32^, ^33^^,^^40^ relied on readability metrics and usability indicators as proxies for comprehension. However, these measures can fall short in assessing whether patients/participants genuinely understand and apply the information presented. Focusing solely on readability rather than comprehension can limit the ability to gauge real-world impact on patient decision making and behavior.^68^ Compounding this issue is the limited involvement of patients/participants in many studies. Without direct patient/participant feedback,^31^ it becomes difficult to determine whether AI-driven applications effectively support health literacy. Furthermore, the small sample sizes and demographically narrow participant groups were seen in some studies,^34^^,^^39^ reducing the generalizability of findings—this is particularly relevant where underserved or disadvantaged populations who might benefit most from improved access to health information, are not considered in the development or implementation of AI in relation to health literacy.

The lack of human review in the included studies highlights an additional limitation; however, it is also important to consider and acknowledge the varying aims of these studies. Some studies focused primarily on assessing the technical performance of AI models, rather than evaluating how such tools would function in real-world settings where human oversight is essential. Without human oversight, health information risks containing inaccuracies, ethical oversights, or content that lacks health relevance.^31^^,^^33^ At the same time, integrating such human review into practice presents its own challenges, including the time and expertise required for detailed review, and the potential for subjective interpretation, which can introduce inconsistencies and bias.^25^ Furthermore, even when human reviewers are included, their attention may diminish over time—especially if AI outputs are mostly accurate—raising questions about the reliability of human-in-the-loop systems. Thus, although human evaluation remains critical, its implementation must be thoughtful, consistent, and well-resourced.

### Limitations of This Review

Our search strategy was limited to selected databases, and English language publications, specified keywords (including “health literacy,” “health education,” and “health promotion”). This may have excluded relevant studies using adjacent concepts such as “readability,” “text simplification,” or “comprehension” without explicitly mentioning health literacy. In addition, given the differing norms of publishing between the health and computing disciplines, our review did not include technical databases (eg, IEEE Xplore), conference proceedings, or preprints, which may have led to the underrepresentation of emerging studies.

### Implications for Future Research

Future research should consider how AI can both enhance the precision of current assessments and support the development of novel approaches that capture the depth and complexity of individuals’ interactions with health information, as well as the organizational contexts and structures that enable or constrain these interactions. Striking a balance between AI automation and human expertise is key—although AI can improve efficiency, human validation ensures reliability and trust in health care communication. Continued efforts are needed to refine AI capabilities to minimize errors while maintaining necessary human involvement for critical oversight. As this is a rapidly evolving field, emerging techniques such as Retrieval-Augmented Generation frameworks can help in addressing limitations by improving the factual accuracy of AI-generated content through integration with trusted external sources. In addition, AI models should integrate context-aware and adaptive responses to enhance human interactions. Moreover, recommending that future studies engage participants with diverse age groups, literacy levels, and cultural backgrounds is only meaningful if we also advance a foundational understanding of how health literacy operates across different populations and contexts. Building this base will ensure that diversity-focused research is not only inclusive but also actionable.

## Conclusion

This systematic review aimed to examine the integration of AI and health literacy, including the definitions of health literacy, the measurement tools used, the AI approaches applied, and the evaluation metrics used. Traditional ML-, deep learning– and transformer-based models were identified, commonly applied to simplify complex medical documents, rewrite materials, and provide interactive access to health information. Health literacy, encompassing both personal and organizational dimensions, is crucial in bridging the gap between health care providers and the public, and the current review highlighted diverse examples of this application in research. The heterogeneity in evaluation methodologies underscores the crucial role of human expertise and the necessity for identifying best practices to ensure accuracy and reliability. Future research should incorporate an evaluation of health literacy that assesses outcomes such as comprehension, decision making, and communication to show effectiveness, balance AI automation with diverse human involvement, and expertise for greater impact.

## Potential Competing Interests

Dr Goss reports institutional grants from 10.13039/501100001601Royal Irish Academy Charlemont Grant, Sunflower Charitable Foundation, Taighde Éireann – Research Ireland, and Pfizer Global Medical Grant—Advancing health literacy in the Republic of Ireland. The other authors declare no conflicts of interest.

## Ethics Statement

Ethical approval was not required for this study as it is a systematic review of previously published literature.

## References

[1] Sørensen K., Van Den Broucke S., Fullam J. (2012). Health literacy and public health: a systematic review and integration of definitions and models. BMC Public Health.

[2] Smith C., Behan S., Belton S., Nicholl C., Murray M., Goss H. (2025). An update on health literacy dimensions: an umbrella review. PLoS One.

[3] World Health Organization (2021).

[4] Jessup R.L., Beauchamp A., Osborne R.H., Hawkins M., Buchbinder R. (2024). Health literacy measurement: a comparison of four widely used health literacy instruments (TOFHLA, NVS, HLS-EU and HLQ) and implications for practice. Aust J Prim Health.

[5] Levin-Zamir D., Leung A.Y.M., Dodson S. (2017). Health literacy in selected populations: Individuals, families, and communities from the international and cultural perspective. Stud Health Technol Inform.

[6] Sørensen K., Pelikan J.M., Röthlin F. (2015). Health literacy in Europe: comparative results of the European health literacy survey (HLS-EU). Eur J Public Health.

[7] Baldwin A.J. (2024). An artificial intelligence language model improves readability of burns first aid information. Burns.

[8] Public Health England; UCL Institute of Health Equity (2015).

[9] Kutner M., Greenberg E., Jin Y., Paulsen C. (2006).

[10] OECD (2013).

[11] Minutolo A., Damiano E., De Pietro G., Fujita H., Esposito M. (2022). A conversational agent for querying Italian Patient Information Leaflets and improving health literacy. Comput Biol Med.

[12] Crossley S.A., Balyan R., Liu J., Karter A.J., McNamara D., Schillinger D. (2020). Predicting the readability of physicians’ secure messages to improve health communication using novel linguistic features: findings from the ECLIPPSE study. J Commun Healthc.

[13] Urstad K.H., Andersen M.H., Larsen M.H., Borge C.R., Helseth S., Wahl A.K. (2022). Definitions and measurement of health literacy in health and medicine research: a systematic review. BMJ Open.

[14] Brega A.G., Hamer M.K., Albright K. (2019). Organizational health literacy: quality improvement measures with expert consensus. Health Lit Res Pract.

[15] Nutbeam D. (2023). Artificial intelligence and health literacy—proceed with caution. Health Lit Commun Open.

[16] Aung Y.Y.M., Wong D.C.S., Ting D.S.W. (2021). The promise of artificial intelligence: a review of the opportunities and challenges of artificial intelligence in healthcare. Br Med Bull.

[17] Ongsulee P. (2017). 2017 15th International Conference on ICT and Knowledge Engineering (ICT&KE).

[18] Almagazzachi A., Mustafa A., Eighaei Sedeh A. (2024). Generative artificial intelligence in patient education: ChatGPT takes on hypertension questions. Cureus.

[19] Sallam M., Mousa D. (2024). Evaluating ChatGPT performance in Arabic dialects: a comparative study showing defects in responding to Jordanian and Tunisian general health prompts. Mesopotamian J Artif Intell Healthc.

[20] Stanceski K., Zhong S., Zhang X. (2024). The quality and safety of using generative AI to produce patient-centred discharge instructions. NPJ Digit Med.

[21] Baglivo F., De Angelis L., Casigliani V., Arzilli G., Privitera G.P., Rizzo C. (2023). Exploring the possible use of AI Chatbots in public health education: feasibility study. JMIR Med Educ.

[22] Guo Y., Liu X., Susarla A., Padman R., Bichel-Findlay J., Otero P., Scott P., Huesing E. (2024). Studies in Health Technology and Informatics.

[23] Will J., Gupta M., Zaretsky J., Dowlath A., Testa P., Feldman J. (2025). Enhancing the readability of online patient education materials using large language models: cross-sectional study. J Med Internet Res.

[24] Nutbeam D., Milat A.J. (2025). Artificial intelligence and public health: prospects, hype and challenges. Public Health Res Pract.

[25] Ali R., Connolly I.D., Tang O.Y. (2024). Bridging the literacy gap for surgical consents: an AI-human expert collaborative approach. NPJ Digit Med.

[26] Caglar U., Yildiz O., Meric A. (2024). Evaluating the performance of ChatGPT in answering questions related to pediatric urology. J Pediatr Urol.

[27] Page M.J., McKenzie J.E., Bossuyt P.M. (2021). The PRISMA 2020 statement: an updated guideline for reporting systematic reviews. BMJ.

[28] Veritas Health Innovation Covidence systematic review software. https://www.covidence.org.

[29] Wen J., Li S., Lin Z., Hu Y., Huang C. (2012). Systematic literature review of machine learning based software development effort estimation models. Inform Softw Technol.

[30] Malhotra R. (2015). A systematic review of machine learning techniques for software fault prediction. Appl Soft Comput.

[31] Sudharshan R., Shen A., Gupta S., Zhang-Nunes S. (2024). Assessing the utility of ChatGPT in simplifying text complexity of patient educational materials. Cureus.

[32] Rouhi A.D., Ghanem Y.K., Yolchieva L. (2024). Can artificial intelligence improve the readability of patient education materials on aortic stenosis? A pilot study. Cardiol Ther.

[33] Kirchner G.J., Kim R.Y., Weddle J.B., Bible J.E. (2023). Can artificial intelligence improve the readability of patient education materials?. Clin Orthop Relat Res.

[34] Hendawi R., Alian S., Li J. (2022). A smart mobile app to simplify medical documents and improve health literacy: system design and feasibility validation. JMIR Form Res.

[35] Ji M., Liu Y., Hao T. (2021). Predicting health material accessibility: development of machine learning algorithms. JMIR Med Inform.

[36] Ji M., Liu Y., Zhao M. (2021). Use of machine learning algorithms to predict the understandability of health education materials: development and evaluation study. JMIR Med Inform.

[37] Mane H.Y., Channell Doig A., Marin Gutierrez F.X. (2023). Practical guidance for the development of Rosie, a health education question-and-answer Chatbot for new mothers. J Public Health Manage Pract.

[38] Yang L.W.Y., Ng W.Y., Lei X. (2023). Development and testing of a multi-lingual natural language processing-based deep learning system in 10 languages for COVID-19 pandemic crisis: a multi-center study. Front Public Health.

[39] Chang Y.H., Guo Y.T., Fu L.C. (2023). Interactive healthcare robot using attention-based question-answer retrieval and medical entity extraction models. IEEE J Biomed Health Inform.

[40] Doppalapudi S., Wang T., Qiu R. (2022). Transforming unstructured digital clinical notes for improved health literacy. Digit Transform Soc.

[41] Sarangi P.K., Lumbani A., Swarup M.S. (2023). Assessing ChatGPT’s proficiency in simplifying radiological reports for healthcare professionals and patients. Cureus.

[42] Vallurupalli M., Shah N.D., Vyas R.M. (2024). Validation of ChatGPT 3.5 as a tool to optimize readability of patient-facing craniofacial education materials. Plast Reconstr Surg Glob Open.

[43] Lalor J.P., Wu H., Chen L., Mazor K.M., Yu H. (2018). ComprehENotes, an instrument to assess patient reading comprehension of electronic health record notes: development and validation. J Med Internet Res.

[44] Hochreiter S., Schmidhuber J. (1997). Long short-term memory. Neural Comput.

[45] Gooding S., Kochmar E. (2019). Proceedings of the 57th Annual Meeting of the Association for Computational Linguistics.

[46] Ma X., Hovy E. (2016). Proceedings of the 54th Annual Meeting of the Association for Computational Linguistics (Volume 1: Long Papers).

[47] Lample G., Ballesteros M., Subramanian S., Kawakami K., Dyer C. (2016). Proceedings of the 2016 Conference of the North American Chapter of the Association for Computational Linguistics: Human Language Technologies.

[48] Kumari V., Gosavi C., Sharma Y., Goel L., Sharma H., Shrivastava V., Kumari Bharti K., Wang L. (2022).

[49] OpenAI, Achiam J., Adler S. (Preprint. Posted online September 19, 2023). GPT-4 technical report. https://arxiv.org/abs/2303.08774.

[50] Raffel C., Shazeer N., Roberts A. (Preprint. Posted online September 19, 2023).

[51] Izacard G., Caron M., Hosseini L. (Preprint. Posted online August 29, 2022).

[52] Vlasov V., Mosig J.E.M., Nichol A. (Preprint. Posted online May 1, 2020).

[53] Wang W., Wei F., Dong L. (Preprint. Posted online April 6, 2020).

[54] Devlin J., Chang M.W., Lee K., Toutanova K. (2019). Proceedings of the 2019 Conference of the North.

[55] Lee J., Yoon W., Kim S. (2020). BioBERT: a pre-trained biomedical language representation model for biomedical text mining. Wren J, ed. Bioinformatics.

[56] Liu Y., Ott M., Goyal N. (Preprint posted online July 26, 2019).

[57] Reimers N., Gurevych I. (2019). Proceedings of the 2019 Conference on Empirical Methods in Natural Language Processing and the 9th International Joint Conference on Natural Language Processing (EMNLP-IJCNLP).

[58] Kulkarni P., Mahabaleshwarkar A., Kulkarni M., Sirsikar N., Gadgil K. (2019). 2019 5th International Conference On Computing, Communication, Control And Automation (ICCUBEA).

[59] Goar V., Yadav N.S., Yadav P.S. (2023). Conversational AI for natural language processing: an review of ChatGPT. Int J Recent Innov Trends Comput Commun.

[60] Cheng Y., Wang D., Zhou P., Zhang T. (2018). Model compression and acceleration for deep neural networks: the principles, progress, and challenges. IEEE Signal Process Mag.

[61] Abibullaev B., Keutayeva A., Zollanvari A. (2023). Deep learning in EEG-based BCIs: a comprehensive review of transformer models, advantages, challenges, and applications. IEEE Access.

[62] Wang P., Fan E., Wang P. (2021). Comparative analysis of image classification algorithms based on traditional machine learning and deep learning. Pattern Recogn Lett.

[63] Kamath C.N., Bukhari S.S., Dengel A. (2018). Proceedings of the ACM Symposium on Document Engineering 2018.

[64] Nasra M., Jaffri R., Pavlin-Premrl D. (2025). Can artificial intelligence improve patient educational material readability? A systematic review and narrative synthesis. Intern Med J.

[65] Pinheiro P. (2021). Conceptualizations of health literacy: past developments, current trends, and possible ways forward toward social practice. Health Lit Res Pract.

[66] Pleasant A. (2014). Advancing health literacy measurement: a pathway to better health and health system performance. J Health Commun.

[67] (2009). Measures of Health Literacy: Workshop Summary.

[68] Singh S., Jamal A., Qureshi F. (2024). Readability metrics in patient education: where do we innovate?. Clin Pract.

