# Supplementary material for: Artificial Intelligence Techniques and Health Literacy: A Systematic Review
Source: Mayo Clin Proc Digit Health. 2025 Sep 24;3(4):100269. doi: 10.1016/j.mcpdig.2025.100269 (PMC12589913; doi:10.1016/j.mcpdig.2025.100269)
Supplement: Supplementary Material [file mmc1.docx]

Table of Contents

[Supplemental Table 1: PRISMA 2020 Checklist 2](#_Toc211491998)

[Supplemental Appendix 1: Keywords and Search Results in Different Databases 5](#_Toc211491999)

[Supplemental Table 2: Demographic Characteristics 9](#_Toc211492000)

[Supplemental Table 3: Glossary 10](#_Toc211492001)

[Supplemental Table 4: Risk of Bias of Selected Studies 12](#_Toc211492002)

# Supplemental Table 1: PRISMA 2020 Checklist

| **Section and Topic** | **Item #** | **Checklist item** | **Location where item is reported** |
| --- | --- | --- | --- |
| **TITLE** | | |  |
| Title | 1 | Identify the report as a systematic review. | 1 |
| **ABSTRACT** | | |  |
| Abstract | 2 | See the PRISMA 2020 for Abstracts checklist. | 2-3 |
| **INTRODUCTION** | | |  |
| Rationale | 3 | Describe the rationale for the review in the context of existing knowledge. | 5-6 |
| Objectives | 4 | Provide an explicit statement of the objective(s) or question(s) the review addresses. | 7 |
| **METHODS** | | |  |
| Eligibility criteria | 5 | Specify the inclusion and exclusion criteria for the review and how studies were grouped for the syntheses. | 8 |
| Information sources | 6 | Specify all databases, registers, websites, organisations, reference lists and other sources searched or consulted to identify studies. Specify the date when each source was last searched or consulted. | 8 |
| Search strategy | 7 | Present the full search strategies for all databases, registers and websites, including any filters and limits used. | Supplemental Appendix 2 |
| Selection process | 8 | Specify the methods used to decide whether a study met the inclusion criteria of the review, including how many reviewers screened each record and each report retrieved, whether they worked independently, and if applicable, details of automation tools used in the process. | 8 |
| Data collection process | 9 | Specify the methods used to collect data from reports, including how many reviewers collected data from each report, whether they worked independently, any processes for obtaining or confirming data from study investigators, and if applicable, details of automation tools used in the process. | 9 |
| Data items | 10a | List and define all outcomes for which data were sought. Specify whether all results that were compatible with each outcome domain in each study were sought (e.g. for all measures, time points, analyses), and if not, the methods used to decide which results to collect. | 9 |
|  | 10b | List and define all other variables for which data were sought (e.g. participant and intervention characteristics, funding sources). Describe any assumptions made about any missing or unclear information. | 9 |
| Study risk of bias assessment | 11 | Specify the methods used to assess risk of bias in the included studies, including details of the tool(s) used, how many reviewers assessed each study and whether they worked independently, and if applicable, details of automation tools used in the process. | 9-10 |
| Effect measures | 12 | Specify for each outcome the effect measure(s) (e.g. risk ratio, mean difference) used in the synthesis or presentation of results. | N/A |
| Synthesis methods | 13a | Describe the processes used to decide which studies were eligible for each synthesis (e.g. tabulating the study intervention characteristics and comparing against the planned groups for each synthesis (item #5)). | N/A |
|  | 13b | Describe any methods required to prepare the data for presentation or synthesis, such as handling of missing summary statistics, or data conversions. | N/A |
|  | 13c | Describe any methods used to tabulate or visually display results of individual studies and syntheses. | 12 |
|  | 13d | Describe any methods used to synthesize results and provide a rationale for the choice(s). If meta-analysis was performed, describe the model(s), method(s) to identify the presence and extent of statistical heterogeneity, and software package(s) used. | 10 |
|  | 13e | Describe any methods used to explore possible causes of heterogeneity among study results (e.g. subgroup analysis, meta-regression). | N/A |
|  | 13f | Describe any sensitivity analyses conducted to assess robustness of the synthesized results. | N/A |
| Reporting bias assessment | 14 | Describe any methods used to assess risk of bias due to missing results in a synthesis (arising from reporting biases). | 9-10 |
| Certainty assessment | 15 | Describe any methods used to assess certainty (or confidence) in the body of evidence for an outcome. | 9-10 |
| **RESULTS** | | |  |
| Study selection | 16a | Describe the results of the search and selection process, from the number of records identified in the search to the number of studies included in the review, ideally using a flow diagram. | 10 |
|  | 16b | Cite studies that might appear to meet the inclusion criteria, but which were excluded, and explain why they were excluded. | N/A |
| Study characteristics | 17 | Cite each included study and present its characteristics. | 34-50 |
| Risk of bias in studies | 18 | Present assessments of risk of bias for each included study. | Supplemental Table 1 |
| Results of individual studies | 19 | For all outcomes, present, for each study: (a) summary statistics for each group (where appropriate) and (b) an effect estimate and its precision (e.g. confidence/credible interval), ideally using structured tables or plots. | N/A |
| Results of syntheses | 20a | For each synthesis, briefly summarise the characteristics and risk of bias among contributing studies. | 17 |
|  | 20b | Present results of all statistical syntheses conducted. If meta-analysis was done, present for each the summary estimate and its precision (e.g. confidence/credible interval) and measures of statistical heterogeneity. If comparing groups, describe the direction of the effect. | N/A |
|  | 20c | Present results of all investigations of possible causes of heterogeneity among study results. | 10-17 |
|  | 20d | Present results of all sensitivity analyses conducted to assess the robustness of the synthesized results. | N/A |
| Reporting biases | 21 | Present assessments of risk of bias due to missing results (arising from reporting biases) for each synthesis assessed. | 17 |
| Certainty of evidence | 22 | Present assessments of certainty (or confidence) in the body of evidence for each outcome assessed. | N/A |
| **DISCUSSION** | | |  |
| Discussion | 23a | Provide a general interpretation of the results in the context of other evidence. | 17-21 |
|  | 23b | Discuss any limitations of the evidence included in the review. | 22-23 |
|  | 23c | Discuss any limitations of the review processes used. | 23 |
|  | 23d | Discuss implications of the results for practice, policy, and future research. | 23-24 |
| **OTHER INFORMATION** | | |  |
| Registration and protocol | 24a | Provide registration information for the review, including register name and registration number, or state that the review was not registered. | 8 |
|  | 24b | Indicate where the review protocol can be accessed, or state that a protocol was not prepared. | 8 |
|  | 24c | Describe and explain any amendments to information provided at registration or in the protocol. | N/A |
| Support | 25 | Describe sources of financial or non-financial support for the review, and the role of the funders or sponsors in the review. | 25 |
| Competing interests | 26 | Declare any competing interests of review authors. | 1 |
| Availability of data, code and other materials | 27 | Report which of the following are publicly available and where they can be found: template data collection forms; data extracted from included studies; data used for all analyses; analytic code; any other materials used in the review. | 25 |

*From:*  Page MJ, McKenzie JE, Bossuyt PM, Boutron I, Hoffmann TC, Mulrow CD, et al. The PRISMA 2020 statement: an updated guideline for reporting systematic reviews. BMJ 2021;372:n71. doi: 10.1136/bmj.n71. This work is licensed under CC BY 4.0. To view a copy of this license, visit <https://creativecommons.org/licenses/by/4.0/>

# Supplemental Appendix 1: Keywords and Search Results in Different Databases

1. **Web of Science**

# Searches:

1: TI=("Machine learning" OR "Artificial intelligence" OR "Deep learning" OR "Neural network" OR "Data Mining" OR ML OR AI OR DM) Timespan: 2014-01-01 to 2024-04-10 Date Run: Wed Apr 10 2024 15:17:48 GMT+0100 (Irish Standard Time) Results: 348383

2: TI=("Health literacy" OR "Health education" OR "Health promotion") Timespan: 2014-01-01 to 2024-04-10 Date Run: Wed Apr 10 2024 15:19:46 GMT+0100 (Irish Standard Time) Results: 16625

3: #2 AND #1 Timespan: 2014-01-01 to 2024-04-10 Date Run: Wed Apr 10 2024 15:19:56 GMT+0100 (Irish Standard Time) Results: 48

4: AB=("Machine learning" OR "Artificial intelligence" OR "Deep learning" OR "Neural network" OR "Data Mining" OR ML OR AI OR DM) Timespan: 2014-01-01 to 2024-04-10 Date Run: Wed Apr 10 2024 15:20:53 GMT+0100 (Irish Standard Time) Results: 1266863

5: AB=("Health literacy" OR "Health education" OR "Health promotion") Timespan: 2014-01-01 to 2024-04-10 Date Run: Wed Apr 10 2024 15:21:07 GMT+0100 (Irish Standard Time) Results: 46646

6: #4 AND #5 Timespan: 2014-01-01 to 2024-04-10 Date Run: Wed Apr 10 2024 15:21:15 GMT+0100 (Irish Standard Time) Results: 846

7: #4 AND #5 and Article (Document Types) Timespan: 2014-01-01 to 2024-04-10 Date Run: Wed Apr 10 2024 15:28:35 GMT+0100 (Irish Standard Time) Results: 710

8: #4 AND #5 and Article (Document Types) and English (Languages) Timespan: 2014-01-01 to 2024-04-10 Date Run: Wed Apr 10 2024 15:29:07 GMT+0100 (Irish Standard Time) Results: 697

9: #2 AND #1 and Article (Document Types) and English (Languages) Timespan: 2014-01-01 to 2024-04-10 Date Run: Wed Apr 10 2024 15:34:35 GMT+0100 (Irish Standard Time) Results: 36

10: #2 AND #1 and Article (Document Types) and English (Languages) and Article (Document Types) Timespan: 2014-01-01 to 2024-04-10 Date Run: Wed Apr 10 2024 15:34:49 GMT+0100 (Irish Standard Time) Results: 36

1. **MEDLINE**

| **#** | **Query** | **Limiters/Expanders** | **Last Run Via** | **Results** |
| --- | --- | --- | --- | --- |
| S6 | (AB "Health literacy" OR  "Health education" OR "Health promotion") AND (S4AND S5) | Expanders - Apply equivalent subjects Search modes - Boolean/Phrase | Interface - EBSCOhost  Research Databases Search Screen - Advanced Search  Database - MEDLINE | 184 |
| S5 | AB ("Health literacy" OR "Health education" OR "Health promotion") | Limiters - Linked Full Text; Peer Reviewed; Publication Date: 20140101-; Abstract Available;  English Language  Expanders - Apply equivalent subjects Search modes - Boolean/Phrase | Interface - EBSCOhost  Research Databases Search Screen - Advanced Search  Database - MEDLINE | 12,220 |
| S4 | AB ( "Machine learning" OR "Artificial intelligence" OR "Deep learning" OR "Neural network" OR  "Data Mining" OR ML) OR AB ( "Machine learning" OR "Artificial intelligence" OR "Deep learning" OR  "Neural network" OR "Data Mining" OR Al ) OR AB ( "Machine learning" OR "Artificial intelligence"  OR "Deep learning" OR "Neural network" OR "Data Mining" OR OM ) | Limiters - Linked Full Text; Peer Reviewed; Publication Date: 20140101-; Abstract Available;  English Language  Expanders - Apply equivalent subjects Search modes - Boolean/Phrase | Interface - EBSCOhost Research Databases Search Screen - Advanced Search  Database - MEDLINE | 118,001 |
| S3 | (Tl "Health literacy" OR "Health education" OR "Health promotion") AND (S1 AND S2) | Expanders - Apply equivalent subjects Search modes - Boolean/Phrase | Interface - EBSCOhost Research Databases Search Screen - Advanced Search  Database - MEDLINE | 7 |
| S2 | Tl ("Health literacy" OR "Health education" OR "Health promotion") | Limiters - Linked Full Text; Peer Reviewed; Publication Date: 20140101-; Abstract | Interface - EBSCOhost  Research Databases Search Screen - Advanced | 2,936 |
| S1 | Tl ( "Machine learning" OR "Artificial intelligence" OR "Deep learning" OR "Neural network" OR  "Data Mining" OR ML) OR Tl ( "Machine learning" OR "Artificial intelligence" OR "Deep learning" OR  "Neural network" OR "Data Mining" OR Al ) OR Tl ( "Machine learning" OR "Artificial intelligence"  OR "Deep learning" OR "Neural network" OR "Data Mining" OR DM ) | Limiters - Linked Full Text; Peer Reviewed; Publication Date: 20140101-; Abstract Available;  English Language  Expanders - Apply equivalent subjects Search modes - Boolean/Phrase | Interface - EBSCOhost Research Databases Search Screen - Advanced Search  Database - MEDLINE | 22,629 |

1. **Scopus**

( TITLE-ABS-KEY ( "Machine learning" OR "Artificial intelligence" OR "Deep learning" OR "Neural network" OR "Data Mining" OR ml OR ai OR dm) AND TITLE­ ABS-KEY ( "Health literacy" OR "Health education" OR "Health promotion")) AND PUBYEAR > 2013 AND ( LIMIT-TO ( SUBJAREA, "HEAL") OR LIMIT-TO ( SUBJAREA, "COMP") OR LIMIT-TO ( SUBJAREA, "SOCI")) AND ( LIMIT-TO ( SRCTYPE, 'T')) AND ( LIMIT-TO ( DOCTYPE, "ar")) AND ( LIMIT-TO (LANGUAGE, "English"))

Results: 336

1. **CINAHL**

XB ( ("Machine learning" OR "Artificial intelligence" OR "Deep learning" OR "Neural network" OR "Data Mining" OR ML OR AI OR DM) ) AND XB ( ("Health literacy" OR "Health education" OR "Health promotion") )

Result: 37

1. **ERIC International**

("Machine learning" OR "Artificial intelligence" OR "Deep learning" OR "Neural network" OR "Data Mining" OR ML OR AI OR DM) AND ("Health literacy" OR "Health education" OR "Health promotion")

Additional limits - Date: From 01 January 2014 to 10 April 2024; Document type: Article; Language: English

Results: 31

1. **PubMed**

(("Machine learning"[Title/Abstract] OR "Artificial intelligence"[Title/Abstract] OR "Deep learning"[Title/Abstract] OR "Neural network"[Title/Abstract] OR "Data Mining"[Title/Abstract] OR ML[Title/Abstract] OR AI[Title/Abstract] OR DM[Title/Abstract]) AND (("Health literacy"[Title/Abstract] OR "Health education"[Title/Abstract] OR "Health promotion"[Title/Abstract])

Results: 793

# Supplemental Table 2: Demographic Characteristics

| No. | Reference, Year | Sample Size | Age | Other |
| --- | --- | --- | --- | --- |
| 1. | Minutolo et al., 2022 | 30 (20 males & 10 females) | 30 - 65 (Average = 49, SD = 12.15) | - |
| 2. | Hendawi et al., 2022 | 28 (13 males & 15 females | 20 – 50 | Education level: 68% undergraduate, 32% postgraduate.  Health literacy level: 36% low, 64% high |
| 3. | Mane et al., 2023 | 109 (females) | - | - |
| 4. | Yang et al., 2023 | 100 | - | - |
| 5. | Chang et al., 2023 | 30 (17 males & 13 females) | 21 – 57 (Average = 27.83, SD = 10.22) | - |

# Supplemental Table 3: Glossary

| BERT (Bidirectional Encoder Representations from Transformers) ^1^ | A language model developed by Google that understands the context of words by looking at both the left and right sides of a sentence. It is widely used in natural language processing tasks. |
| --- | --- |
| BiLSTM-CNN-CRF ^2^ | A hybrid model combining Bidirectional Long Short-Term Memory (BiLSTM), Convolutional Neural Networks (CNN), and Conditional Random Fields (CRF) for tasks like named entity recognition. |
| BioBERT ^3^ | A version of BERT pre-trained on large biomedical corpora such as PubMed, designed for biomedical text mining. |
| Classification models | A type of machine learning methods used to assign inputs (such as text or images) into predefined categories. For example, a model may classify emails as “spam” or “not spam.” |
| Contriever ^4^ | A retrieval model developed by Meta AI that retrieves relevant documents from large text corpora based on learned representations. |
| Decision Tree | A model that makes decisions by splitting data into branches based on feature values, often used for classification. |
| GPT-4 (Generative Pretrained Transformer 4) ^5^ | A large multimodal language model developed by OpenAI capable of generating and understanding human-like text. It is used for tasks such as summarization, translation, and dialogue. |
| Information retrieval | The process of searching for and obtaining relevant information from a large collection of data sources, such as retrieving articles or answers from medical databases. |
| K-Nearest Neighbors (KNN) | A model that classifies data based on the majority label of its nearest neighbours in the feature space. |
| Linear Discriminant Analysis (LDA) | A method used for dimensionality reduction and classification, which finds a linear combination of features that best separates two or more classes. |
| LogitBoost | A boosting algorithm that improves classification performance by combining multiple weak learners into a strong one, especially useful for binary classification. |
| LSTM (Long Short-Term Memory) ^6^ | A type of recurrent neural network (RNN) that is capable of learning long-term dependencies in sequence data, often used in language modelling. |
| Machine learning metrics | Simple tools or numbers used to measure how well an AI or machine learning model is doing. They help researchers understand if the model is making accurate predictions, providing useful answers, or producing understandable results. |
| Medical entity identification | A natural language processing task that detects and labels medically relevant terms (e.g., diseases, drugs, symptoms) within text. |
| MiniLM ^7^ | A lightweight and faster version of BERT, designed to provide competitive performance with fewer parameters, making it suitable for resource-constrained environments. |
| Multilayer Perceptron (MLP) | A basic form of neural network with multiple layers of nodes, useful for tasks like classification and regression. |
| Question encoding | The process of converting a question into a numerical representation so that it can be compared with other text or used by machine learning models for tasks such as search or retrieval. |
| Random Forest | An ensemble learning method that combines multiple decision trees to improve predictive accuracy and control overfitting. |
| RoBERTa (Robustly Optimized BERT Pretraining Approach) ^8^ | A modified version of BERT by Meta, trained with more data and longer sequences for better language understanding. |
| SBERT (Sentence-BERT) ^9^ | A modification of BERT that produces sentence embeddings, allowing for efficient comparison of sentence similarity. |
| Semantic search | A search approach that goes beyond matching keywords by considering the meaning and context of words, enabling systems to recognize that terms such as “heart attack” and “myocardial infarction” are equivalent. |
| Sequence Labelling Model ^10^ | A model that assigns labels to each element in a sequence, often used in named entity recognition and part-of-speech tagging. |
| spaCy Library | An open-source software library for advanced natural language processing in Python, often used for tokenization, parsing, and entity recognition. |
| Support Vector Machine (SVM) | A classification algorithm that finds the best boundary (hyperplane) to separate different classes of data. |
| T5 (Text-to-Text Transfer Transformer) ^11^ | A model by Google that treats NLP tasks as a text-to-text problem, making it versatile for tasks like summarization, question answering, and translation. |
| Transformer Embedding Dialogue Model ^12^ | A transformer-based model designed to embed and understand multi-turn dialogues, often used in conversational AI systems |

#

# Supplemental Table 4: Risk of Bias of Selected Studies

| **No.** | **Reference, year** | **Q1** | **Q2** | **Q3** | **Q4** | **Q5** | **Q6** | **Q7** | **Q8** | **Q9** | **Q10** | **Q11** | **Q12** | **Q13** | **Q14** | **Score** |
| --- | --- | --- | --- | --- | --- | --- | --- | --- | --- | --- | --- | --- | --- | --- | --- | --- |
| 1. | Ali et al., 2024 | 1 | 1 | 0.5 | 1 | 0 | 1 | 0 | 1 | 1 | 0 | 0.5 | 0 | 0 | 0 | **7** |
| 2. | Almagazzachi et al., 2024 | 1 | 1 | 0.5 | 0.5 | 0 | 1 | 1 | 1 | 1 | 1 | 0.5 | 0 | 0 | 0 | **8.5** |
| 3. | Baldwin, 2024 | 1 | 1 | 0.5 | 0.5 | 0 | 1 | 1 | 1 | 1 | 1 | 0.5 | 0 | 1 | 0 | **9.5** |
| 4. | Caglar et al., 2024 | 1 | 1 | 0.5 | 1 | 0 | 1 | 1 | 1 | 1 | 1 | 0.5 | 0 | 1 | 0 | **10** |
| 5. | Chang et al., 2023 | 1 | 1 | 0.5 | 1 | 1 | 1 | 1 | 1 | 1 | 0.5 | 0.5 | 1 | 0 | 0 | **10.5** |
| 6. | Crossley et al., 2020 | 1 | 1 | 0.5 | 1 | 0 | 1 | 1 | 1 | 1 | 1 | 0.5 | 1 | 1 | 0 | **11** |
| 7. | Doppalapudi et al., 2022 | 1 | 1 | 1 | 1 | 0 | 1 | 1 | 1 | 1 | 1 | 0.5 | 0 | 1 | 0 | **10.5** |
| 8. | Hendawi et al., 2022 | 1 | 1 | 0 | 0.5 | 1 | 1 | 1 | 1 | 1 | 1 | 0.5 | 0 | 1 | 1 | **11** |
| 9. | Ji et al., 2021 | 1 | 1 | 0.5 | 0.5 | 0 | 1 | 1 | 1 | 1 | 1 | 0.5 | 1 | 0 | 0 | **9.5** |
| 10. | Ji et al., 2021 | 1 | 1 | 0.5 | 0.5 | 0 | 1 | 1 | 1 | 1 | 1 | 0.5 | 1 | 0 | 0 | **9.5** |
| 11. | Kirchner et al., 2023 | 1 | 0.5 | 0 | 0.5 | 0 | 1 | 1 | 1 | 1 | 1 | 0.5 | 0 | 0 | 0 | **7.5** |
| 12. | Mane et al., 2023 | 1 | 1 | 0.5 | 1 | 1 | 1 | 1 | 0.5 | 1 | 1 | 0.5 | 1 | 0 | 0 | **10.5** |
| 13. | Minutolo et al., 2022 | 1 | 1 | 0.5 | 0.5 | 1 | 1 | 1 | 1 | 1 | 0.5 | 0.5 | 0 | 1 | 0 | **10** |
| 14. | Rouhi et al., 2024 | 1 | 1 | 0.5 | 0.5 | 0 | 1 | 1 | 1 | 1 | 1 | 0.5 | 1 | 0 | 0 | **9.5** |
| 15. | Sarangi et al., 2023 | 1 | 1 | 0.5 | 0.5 | 0 | 1 | 1 | 1 | 1 | 1 | 0.5 | 0 | 0 | 0 | **7.5** |
| 16. | Sudharshan et al., 2024 | 1 | 1 | 0.5 | 0.5 | 0 | 1 | 1 | 1 | 1 | 1 | 0.5 | 0 | 0 | 0 | **8.5** |
| 17. | Vallurupalli et al., 2024 | 1 | 1 | 0.5 | 0.5 | 0 | 1 | 1 | 1 | 1 | 1 | 0.5 | 0 | 1 | 0 | **9.5** |
| 18. | Yang et al., 2023 | 1 | 1 | 1 | 1 | 1 | 1 | 1 | 1 | 1 | 1 | 0.5 | 0 | 0 | 0 | **10.5** |

**Supplemental References**

1. Devlin J, Chang MW, Lee K, Toutanova K. BERT: Pre-training of Deep Bidirectional Transformers for Language Understanding. In: *Proceedings of the 2019 Conference of the North*. Association for Computational Linguistics; 2019:4171-4186. doi:10.18653/v1/N19-1423

2. Ma X, Hovy E. End-to-end Sequence Labeling via Bi-directional LSTM-CNNs-CRF. In: *Proceedings of the 54th Annual Meeting of the Association for Computational Linguistics (Volume 1: Long Papers)*. Association for Computational Linguistics; 2016:1064-1074. doi:10.18653/v1/P16-1101

3. Lee J, Yoon W, Kim S, et al. BioBERT: a pre-trained biomedical language representation model for biomedical text mining. Wren J, ed. *Bioinformatics*. 2020;36(4):1234-1240. doi:10.1093/bioinformatics/btz682

4. Izacard G, Caron M, Hosseini L, et al. Unsupervised Dense Information Retrieval with Contrastive Learning. *Transactions on Machine Learning Research*. Published online 2022. Accessed March 17, 2025. https://arxiv.org/abs/2112.09118

5. OpenAI, Achiam J, Adler S, et al. GPT-4 Technical Report. Published online 2023. Accessed March 17, 2025. https://arxiv.org/abs/2303.08774

6. Hochreiter S, Schmidhuber J. Long Short-Term Memory. *Neural Computation*. 1997;9(8):1735-1780. doi:10.1162/neco.1997.9.8.1735

7. Wang W, Wei F, Dong L, Bao H, Yang N, Zhou M. MiniLM: Deep Self-Attention Distillation for Task-Agnostic Compression of Pre-Trained Transformers. Published online 2020. doi:10.48550/ARXIV.2002.10957

8. Liu Y, Ott M, Goyal N, et al. RoBERTa: A Robustly Optimized BERT Pretraining Approach. Published online July 26, 2019. doi:10.48550/arXiv.1907.11692

9. Reimers N, Gurevych I. Sentence-BERT: Sentence Embeddings using Siamese BERT-Networks. In: *Proceedings of the 2019 Conference on Empirical Methods in Natural Language Processing and the 9th International Joint Conference on Natural Language Processing (EMNLP-IJCNLP)*. Association for Computational Linguistics; 2019:3980-3990. doi:10.18653/v1/D19-1410

10. Gooding S, Kochmar E. Complex Word Identification as a Sequence Labelling Task. In: *Proceedings of the 57th Annual Meeting of the Association for Computational Linguistics*. Association for Computational Linguistics; 2019:1148-1153. doi:10.18653/v1/P19-1109

11. Raffel C, Shazeer N, Roberts A, et al. Exploring the Limits of Transfer Learning with a Unified Text-to-Text Transformer. *Journal of Machine Learning Research*. 2020;21. Accessed March 17, 2025. https://arxiv.org/abs/1910.10683

12. Vlasov V, Mosig JEM, Nichol A. Dialogue Transformers. Published online May 1, 2020. Accessed March 18, 2025. http://arxiv.org/abs/1910.00486
